# Fourier series of higher-order Bernoulli functions and their applications

**DOI:** 10.1186/s13660-016-1282-y

**Published:** 2017-01-05

**Authors:** Taekyun Kim, Dae San Kim, Seog-Hoon Rim, Dmitry V. Dolgy

**Affiliations:** 1 Department of Mathematics, Kwangwoon University, Seoul, 139-701 Republic of Korea; 2 Department of Mathematics, College of Science, Tianjin Polytechnic University, Tianjin, 300160 China; 3 Department of Mathematics, Sogang University, Seoul, 121-742 Republic of Korea; 4 Department of Mathematics Education, Kyungpook National University, Taegu, 702-701 Republic of Korea; 5 Hanrimwon, Kwangwoon University, Seoul, 139-701 Republic of Korea; 6 School of Natural Sciences, Far Eastern Federal University, 690950 Vladivostok, Russia

**Keywords:** 11B68, 42A16, Fourier series, Bernoulli polynomials, Bernoulli functions

## Abstract

In this paper, we study the Fourier series related to higher-order Bernoulli functions and give new identities for higher-order Bernoulli functions which are derived from the Fourier series of them.

## Introduction

As is well known, Bernoulli polynomials are defined by the generating function

1.1 $$ \begin{aligned} \frac{t}{e^{t}-1}e^{xt} = \sum _{n=0}^{\infty}B_{n}(x) \frac{t^{n}}{n!}\quad (\mbox{see [1--23]}). \end{aligned} $$

When $x=0$, $B_{n}=B_{n}(0)$ are called Bernoulli numbers. From (1.1), we note that

1.2 $$ \begin{aligned} B_{n}(x) = \sum _{l=0}^{n} {n \choose l} B_{l} x^{n-l} \in\mathbb{Q}[x]\quad (n \geq0), \end{aligned} $$

with $\deg B_{n}(x)=n$ (see [9–11]). By (1.1), we easily get

1.3 $$ \begin{aligned} (B+1)^{n} - B_{n} = \textstyle\begin{cases} 1,&\text{if } n=1,\\ 0,&\text{if }n>1, \end{cases}\displaystyle \quad\text{and}\quad B_{0}=1, \end{aligned} $$

with the usual convention about replacing $B^{n}$ by $B_{n}$ (see [9, 10]). From (1.2), we note that

1.4 $$ \begin{aligned}[b] \frac{dB_{n}(x)}{dx} &=\frac{d}{dx}\sum _{k=0}^{n} {n \choose k}B_{k}x^{n-k} = \sum_{k=0}^{n-1} {n \choose k}B_{k}(n-k) x^{n-k-1} \\ &=n \sum_{k=0}^{n-1} \frac{(n-1)!}{( n-k-1)!k!} B_{k} x^{n-k-1} = n \sum_{k=0}^{n-1} {n-1 \choose k}B_{k} x^{n-1-k} \\ &= nB_{n-1}(x) \quad (n \geq1)\ (\mbox{see [9--18]}). \end{aligned} $$

Thus, by (1.4), we get

1.5 $$ \begin{aligned} \int_{0}^{x} B_{n}(x)\,dx = \frac{1}{n+1} \bigl( B_{n+1}(x) - B_{n+1}(0) \bigr) \quad (n \geq0). \end{aligned} $$

For any real number *x*, we define

1.6 $$ \begin{aligned} \langle x \rangle= x-[x] \in[0,1), \end{aligned} $$

where $[x]$ is the integral part of *x*. Then $B_{n}( \langle x \rangle)$ are functions defined on $(-\infty, \infty)$ and periodic with period 1, which are called Bernoulli functions. The Fourier series for $B_{m}( \langle x \rangle)$ is given by

1.7 $$ \begin{aligned} B_{m}\bigl( \langle x \rangle \bigr) = -m! \sum_{\substack{n=-\infty\\n \neq0}}^{\infty}\frac{e^{2\pi inx}}{(2 \pi in)^{m}}, \end{aligned} $$

where $m \geq1$ and $x \notin\mathbb{Z}$ (see [1, 2, 8, 14, 22]). For a positive integer N, we have

$$ \begin{aligned} \sum_{k=0}^{N-1}B_{m} \biggl( \biggl\langle \frac{x+k}{N} \biggr\rangle \biggr) &=-m! \sum _{k=0}^{N-1} \sum _{\substack{n=-\infty\\n \neq0}}^{\infty}\frac{e^{2\pi in (\frac{x+k}{N} ) }}{(2 \pi in)^{m}} \\ &=-m!\sum_{\substack{n=-\infty\\n \neq0}}^{\infty}\frac{e^{2\pi in \frac {x}{N} }}{(2 \pi in)^{m}} \sum _{k=0}^{N-1} e^{2 \pi in \frac{k}{N}} \\ &=-m! N^{1-m} \sum_{\substack{l=-\infty\\l \neq0}}^{\infty}\frac {e^{2\pi il x }}{(2 \pi il)^{m}} = N^{1-m} B_{m}\bigl( \langle x \rangle \bigr) \quad (x \notin\mathbb{Z}). \end{aligned} $$

For $r \in\mathbb{N}$, the higher-order Bernoulli polynomials are defined by the generating function

1.8 $$ \begin{aligned} \biggl( \frac{t}{e^{t}-1} \biggr)^{r} e^{xt} = \sum_{n=0}^{\infty}B_{n}^{(r)}(x) \frac{t^{n}}{n!}\quad(\mbox{see [1, 10, 11, 22]}). \end{aligned} $$

When $x=0$, $B_{n}^{(r)}= B_{n}^{(r)}(0)$ are called Bernoulli numbers of order *r* (see [1, 22]). Then $B_{n}^{(r)}( \langle x \rangle)$ are functions defined on $(-\infty, \infty)$ and periodic with period 1, which are called Bernoulli functions of order *r*. In this paper, we study the Fourier series related to higher-order Bernoulli functions and give some new identities for the higher-order Bernoulli functions which are derived from the Fourier series of them.

## Fourier series of higher-order Bernoulli functions and their applications

From (1.8), we note that

2.1 $$ \begin{aligned} B_{m}^{(r)}(x+1) = B_{m}^{(r)}(x) + m B_{m-1}^{(r-1)}(x) \quad (m \geq0). \end{aligned} $$

Indeed,

2.2 $$ \begin{aligned}[b] \sum_{m=0}^{\infty}B_{m}^{(r)}(x+1) \frac{t^{m}}{m!} &= \biggl( \frac {t}{e^{t}-1} \biggr)^{r} e^{(x+1)t}= \biggl( \frac{t}{e^{t}-1} \biggr)^{r} e^{xt}\bigl(e^{t} -1 +1\bigr) \\ &= \biggl( \frac{t}{e^{t}-1} \biggr)^{r-1} te^{xt}+ \biggl( \frac{t}{e^{t}-1} \biggr)^{r} e^{xt} \\ &=\sum_{m=0}^{\infty}B_{m}^{(r-1)}(x) \frac{t^{m+1}}{m!} + \sum_{m=0}^{\infty}B_{m}^{(r)}(x) \frac{t^{m}}{m!} \\ &= \sum_{m=0}^{\infty}\bigl( m B_{m-1}^{(r-1)}(x) + B_{m}^{(r)}(x) \bigr) \frac{t^{m}}{m!}. \end{aligned} $$

Let $x=0$ in (2.1). Then we have

2.3 $$ \begin{aligned} B_{m}^{(r)}(1) = B_{m}^{(r)}(0) + mB_{m-1}^{(r-1)}(0)\quad ( m \geq0). \end{aligned} $$

Now, we assume that $m \geq1$, $r \geq2$. $B_{m}^{(r)}( \langle x \rangle)$ is piecewise $C^{\infty}$. Further, in view of (2.3), $B_{m}^{(r)}( \langle x \rangle)$ is continuous for those $(r,m)$ with $B_{m-1}^{(r-1)}(0) =0$, and is discontinuous with jump discontinuities at integers for those $(r,m)$ with $B_{m-1}^{(r-1)}(0) \neq0$. The Fourier series of $B_{m}^{(r)}( \langle x \rangle )$ is

2.4 $$ \begin{aligned} \sum_{n=-\infty}^{\infty}C_{n}^{(r,m)}e^{2 \pi inx}\quad (i = \sqrt{-1}), \end{aligned} $$

where

2.5 $$ \begin{aligned}[b] C_{n}^{(r,m)} &= \int_{0}^{1} B_{m}^{(r)}\bigl( \langle x \rangle\bigr) e^{-2 \pi inx} \,dx = \int_{0}^{1} B_{m}^{(r)}(x) e^{-2 \pi inx}\,dx \\ &= \biggl[ \frac{1}{m+1} B_{m+1}^{(r)}(x) e^{-2 \pi inx} \biggr]_{0}^{1} + \frac{2 \pi in}{m+1} \int_{0}^{1} B_{m+1}^{(r)}(x) e^{-2 \pi inx} \,dx \\ &= \frac{1}{m+1} \bigl( B_{m+1}^{(r)}(1) - B_{m+1}^{(r)}(0) \bigr) + \frac{2 \pi in}{m+1} C_{n}^{(r,m+1)} \\ &= B_{m}^{(r-1)}(0) + \frac{2 \pi in}{m+1} C_{n}^{(r,m+1)}. \end{aligned} $$

Replacing *m* by $m-1$ in (2.5), we get

2.6 $$ \begin{aligned} C_{n}^{(r,m-1)} = B_{m-1}^{(r-1)}(0) + \frac{2 \pi in}{m} C_{n}^{(r,m)}. \end{aligned} $$

Case 1. Let $n \neq0$. Then we have

2.7 $$\begin{aligned} C_{n}^{(r,m)}&= \frac{m}{2 \pi in } C_{n}^{(r,m-1)}- \frac{m}{2\pi in}B_{m-1}^{(r-1)} \\ &= \frac{m}{2\pi in} \biggl( \frac{m-1}{2\pi in} C_{n}^{(r,m-2)}- \frac {m-1}{2\pi in} B_{m-2}^{(r-1)} \biggr) - \frac{m}{2\pi in}B_{m-1}^{(r-1)} \\ &= \frac{m(m-1)}{(2\pi in)^{2}}C_{n}^{(r,m-2)} - \frac{m(m-1)}{(2\pi in)^{2}}B_{m-2}^{(r-1)} - \frac{m}{2\pi in}B_{m-1}^{(r-1)} \\ &=\frac{m(m-1)}{(2\pi in)^{2}} \biggl\{ \frac{m-2}{2\pi in} C_{n}^{(r,m-3)}- \frac{m-2}{2\pi in} B_{m-3}^{(r-1)} \biggr\} - \frac {m(m-1)}{(2\pi in)^{2}}B_{m-2}^{(r-1)} - \frac{m}{2\pi in}B_{m-1}^{(r-1)} \\ &= \frac{m(m-1)(m-2)}{(2\pi in)^{3}} C_{n}^{(r,m-3)} - \frac {m(m-1)(m-2)}{(2\pi in)^{3}} B_{m-3}^{(r-1)} \\ &\quad {} - \frac{m(m-1)}{(2\pi in)^{2}}B_{m-2}^{(r-1)} - \frac{m}{2\pi in}B_{m-1}^{(r-1)} \\ &= \cdots \\ &= \frac{m(m-1)(m-2)\cdots2}{(2\pi in)^{m-1}} C_{n}^{(r,1)} - \sum _{k=1}^{m-1} \frac{(m)_{k}}{(2\pi in)^{k}} B_{m-k}^{(r-1)}, \end{aligned}$$

where $(x)_{n} = x(x-1)\cdots(x-n+1)$, for $n \geq1$, and $(x)_{0}=1$. Now, we observe that

2.8 $$ \begin{aligned}[b] C_{n}^{(r,1)} &= \int_{0}^{1} B_{1}^{(r)}(x) e^{-2 \pi inx}\,dx = \int_{0}^{1} \bigl(x+B_{1}^{(r)} \bigr) e^{-2 \pi inx} \,dx \\ &= \int_{0}^{1} xe^{-2 \pi inx}\,dx + B_{1}^{(r)} \int_{0}^{1} e^{-2 \pi inx}\,dx \\ &= - \frac{1}{2 \pi in} \bigl[ x e^{-2 \pi inx} \bigr]_{0}^{1} + \frac {1}{2 \pi in} \int_{0}^{1} e^{-2 \pi inx} \,dx \\ &= - \frac{1}{2 \pi in}. \end{aligned} $$

From (2.7) and (2.8), we can derive equation (2.9):

2.9 $$ \begin{aligned}[b] C_{n}^{(r,m)} &= - \frac{m!}{(2 \pi in)^{m}} - \sum_{k=1}^{m-1} \frac {(m)_{k}}{(2 \pi in)^{k}} B_{m-k}^{(r-1)} \\ &= - \sum_{k=1}^{m} \frac {(m)_{k}}{(2 \pi in)^{k}}B_{m-k}^{(r-1)}. \end{aligned} $$

Case 2. Let $n=0$. Then we have

2.10 $$ \begin{aligned}[b] C_{0}^{(r,m)} &= \int_{0}^{1} B_{m}^{(r)}\bigl( \langle x \rangle\bigr) \,dx = \int_{0}^{1} B_{m}^{(r)}(x) \,dx = \frac{1}{m+1} \bigl[ B_{m+1}^{(r)}(x) \bigr]_{0}^{1} \\ &= \frac{1}{m+1} \bigl( B_{m+1}^{(r)}(1) - B_{m+1}^{(r)}(0) \bigr) = B_{m}^{(r-1)}. \end{aligned} $$

Before proceeding, we recall the following equations:

2.11 $$ \begin{aligned} B_{m}\bigl( \langle x \rangle \bigr) = -m! \sum_{\substack{n=-\infty\\ n \neq0}}^{\infty}\frac{e^{2 \pi inx}}{(2 \pi in)^{m}}, \quad (m \geq2) \ (\mbox{see [1]}), \end{aligned} $$

and

2.12 $$ \begin{aligned} -\sum_{\substack{n=-\infty\\ n \neq0}}^{\infty}\frac{e^{2 \pi inx}}{2 \pi in} = \textstyle\begin{cases} B_{1}( \langle x \rangle),& \text{for } x \notin\mathbb {Z},\\ 0,& \text{for } x \in\mathbb{Z},\ (\mbox{see [1, 22]}). \end{cases}\displaystyle \end{aligned} $$

The series in (2.11) converges uniformly, while that in (2.12) converges pointwise. Assume first that $B_{m-1}^{(r-1)}(0) =0$. Then we have $B_{m}^{(r)}(1) = B_{m}^{(r)}(0)$, and $m \geq2$. As $B_{m}^{(r)}( \langle x \rangle)$ is piecewise $C^{\infty}$ and continuous, the Fourier series of $B_{m}^{(r)}( \langle x \rangle)$ converges uniformly to $B_{m}^{(r)}( \langle x \rangle)$, and

2.13 $$ \begin{aligned}[b] B_{m}^{(r)}\bigl( \langle x \rangle\bigr) &= \sum_{n=-\infty}^{\infty}C_{n}^{(r,m)}e^{2 \pi inx} \\ &= B_{m}^{(r-1)} - \sum_{\substack{n=-\infty\\ n \neq0}}^{\infty}\Biggl(\sum_{k=1}^{m} \frac{(m)_{k}}{(2 \pi in)^{k}}B_{m-k}^{(r-1)} \Biggr) e^{2 \pi inx} \\ &= B_{m}^{(r-1)} + \sum_{k=1}^{m} \frac{(m)_{k}}{k!} B_{m-k}^{(r-1)} \cdot \Biggl( -k! \sum _{\substack{n=-\infty\\ n \neq0}}^{\infty}\frac{e^{2 \pi inx}}{(2 \pi in)^{k}} \Biggr) \\ &= B_{m}^{(r-1)} + \sum_{k=2}^{m} {m \choose k} B_{m-k}^{(r-1)} B_{k} \bigl( \langle x \rangle\bigr) \\ &\quad{}+ {m \choose 1} B_{m-1}^{(r-1)} \times \textstyle\begin{cases} B_{1}( \langle x \rangle),& \text{for } x \notin\mathbb {Z},\\ 0,& \text{for } x \in\mathbb{Z} \end{cases}\displaystyle \\ &= \textstyle\begin{cases} \sum_{k=0}^{m} {m \choose k} B_{m-k}^{(r-1)}B_{k}( \langle x \rangle) & \text{for } x \notin\mathbb{Z},\\ \sum_{\substack{k=0\\k \neq1}}^{m} {m \choose k} B_{m-k}^{(r-1)}B_{k}( \langle x \rangle) & \text{for } x \in\mathbb{Z}. \end{cases}\displaystyle \end{aligned} $$

Note that (2.13) holds whether $B_{m-1}^{(r-1)}(0)=0$ or not. However, if $B_{m-1}^{(r-1)}(0)=0$, then

$$ \begin{aligned} B_{m}^{(r)}\bigl( \langle x \rangle \bigr) = \sum_{\substack{k=0 \\ k \neq 1}}^{m} {m \choose k} B_{m-k}^{(r-1)} B_{k} \bigl( \langle x \rangle\bigr), \quad \text{for all } x \in(-\infty, \infty). \end{aligned} $$

Therefore, we obtain the following theorem.

### Theorem 2.1

*Let* $m \geq2$, $r \geq2$. *Assume that* $B_{m-1}^{(r-1)}(0)=0$.

- $B_{m}^{(r)}( \langle x \rangle)$ *has the Fourier series expansion*
  $$ \begin{aligned} B_{m}^{(r)}\bigl( \langle x \rangle \bigr) = B_{m}^{(r-1)}(0) - \sum_{\substack{n=-\infty\\ n \neq0}}^{\infty}\Biggl(\sum_{k=1}^{m} \frac {(m)_{k}}{(2 \pi in)^{k}}B_{m-k}^{(r-1)} \Biggr) e^{2 \pi inx}, \end{aligned} $$
  *for* $x \in(-\infty, \infty)$. *Here the convergence is uniform*.
- $B_{m}^{(r)}( \langle x \rangle) = \sum_{\substack{k=0 \\ k \neq1}}^{m} {m \choose k} B_{m-k}^{(r-1)} B_{k} ( \langle x \rangle)$, *for all* $x \in(-\infty, \infty)$, *where* $B_{k}( \langle x \rangle)$ *is the Bernoulli function*.

Assume next that $B_{m-1}^{(r-1)}(0) \neq0$. Then $B_{m}^{(r)}(1) \neq B_{m}^{(r)}(0)$, and hence $B_{m}^{(r)}( \langle x \rangle)$ is piecewise $C^{\infty}$ and discontinuous with jump discontinuities at integers Thus the Fourier series of $B_{m}^{(r)}( \langle x \rangle)$ converges pointwise to $B_{m}^{(r)}( \langle x \rangle)$, for $x \notin\mathbb{Z}$, and converges to $\frac{1}{2} ( B_{m}^{(r)}(0) + B_{m}^{(r)}(1) ) = B_{m}^{(r)}(0) + \frac{m}{2} B_{m-1}^{(r-1)}(0)$, for $x \in\mathbb{Z}$. Thus we obtain the following theorem.

### Theorem 2.2

*Let* $m \geq1$, $r \geq2$, *Assume that* $B_{m-1}^{(r-1)}(0) \neq0$.

$$ \begin{aligned} (\mathrm{a}) \quad B_{m}^{(r-1)}(0) - \sum_{\substack{n=-\infty\\ n \neq0}}^{\infty}\Biggl(\sum _{k=1}^{m} \frac{(m)_{k}}{(2 \pi in)^{k}}B_{m-k}^{(r-1)} \Biggr) e^{2 \pi inx} = \textstyle\begin{cases} B_{m}^{(r)}( \langle x \rangle),& \textit{for } x \notin \mathbb{Z},\\ B_{m}^{(r)} + \frac{m}{2} B_{m-1}^{(r-1)},& \textit{for } x \in\mathbb{Z}. \end{cases}\displaystyle \end{aligned} $$

*Here the convergence is pointwise*,

$$ \begin{aligned} (\mathrm{b}) \quad \sum_{k=0}^{m} {m \choose k} B_{m-k}^{(r-1)} B_{k} \bigl( \langle x \rangle\bigr) = B_{m}^{(r)}(x), \quad \textit{for } x \notin\mathbb{Z}, \end{aligned} $$

*and*

$$ \begin{aligned} \sum_{\substack{k=0 \\ k \neq1}}^{m} {m \choose k} B_{m-k}^{(r-1)} B_{k} \bigl( \langle x \rangle\bigr) = B_{m}^{(r)} + \frac{m}{2} B_{m-1}^{(r-1)}, \quad \textit{for } x \in\mathbb{Z}, \end{aligned} $$

*where* $B_{k}( \langle x \rangle)$ *is the Bernoulli function*.

### Remark

Let $\zeta(s) = \sum_{n=1}^{\infty}\frac{1}{n^{s}}$, $( \operatorname{Re}(s)>1 )$. From (1.7), we note that, for $m \geq1$,

$$ \begin{aligned} B_{2m} &= -(2m)! \sum _{\substack{n=-\infty\\n \neq0}}^{\infty}\frac {(-1)^{m}}{(2 \pi n)^{2m}} \\ &= - \frac{(2m)!}{(2\pi)^{2m}} 2 \sum_{n=1}^{\infty}\frac {(-1)^{m}}{n^{2m}} = (-1)^{m+1} \frac{2(2m)!}{(2\pi)^{2m}} \zeta(2m). \end{aligned} $$

## Results and discussion

In this paper, we studied the Fourier series expansion of the higher-order Bernoulli functions $B_{m}^{(r)}( \langle x \rangle )$ which are obtained by extending by periodicity of period 1 the higher-order Bernoulli polynomials $B_{m}^{(r)}(x)$ on $[0, 1)$. As it turns out, the Fourier series of $B_{m}^{(r)}( \langle x \rangle)$ converges uniformly to $B_{m}^{(r)}( \langle x \rangle)$, if $B_{m-1}^{(r-1)}(0)=0$, and converges pointwise to $B_{m}^{(r)}( \langle x \rangle)$ for $x\notin \Bbb {Z}$ and converges to $B_{m}^{(r)}+\frac{m}{2}B_{m-1}^{(r-1)}$ for $x\in\Bbb {Z}$, if $B_{m-1}^{(r-1)}(0)\neq0$. Here the Fourier series of the higher-order Bernoulli functions $B_{m}^{(r)}( \langle x \rangle)$ are explicitly determined. In addition, in each case the Fourier series of the higher-order Bernoulli functions $B_{m}^{(r)}( \langle x \rangle)$ are expressed in terms of Bernoulli functions which are obtained by extending by periodicity of period 1 the ordinary Bernoulli polynomials $B_{m}(x)$ on $[0, 1)$. The Fourier series expansion of the Bernoulli functions are useful in computing the special values of the Dirichlet *L*-functions. For details, one is referred to [24].

It is expected that the Fourier series of the higher-order Bernoulli functions will find some applications in connections with a certain generalization of Dirichlet *L*-functions and higher-order generalized Bernoulli numbers.

## Conclusion

In this paper, we considered the Fourier series expansion of the higher-order Bernoulli functions $B_{m}^{(r)}( \langle x \rangle )$ which are obtained by extending by periodicity of period 1 the higher-order Bernoulli polynomials $B_{m}^{(r)}(x)$ on $[0, 1)$. The Fourier series are explicitly determined. Depending on whether $B_{m-1}^{(r-1)}(0)$ is zero or not, the Fourier series of $B_{m}^{(r)}( \langle x \rangle)$ converges uniformly to $B_{m}^{(r)}( \langle x \rangle)$ or converges pointwise to $B_{m}^{(r)}( \langle x \rangle)$ for $x\notin\Bbb {Z}$ and converges to $B_{m}^{(r)}+\frac {m}{2}B_{m-1}^{(r-1)}$ for $x\in\Bbb {Z}$. In addition, the Fourier series of the higher-order Bernoulli functions $B_{m}^{(r)}( \langle x \rangle)$ are expressed in terms of Bernoulli functions $B_{k}( \langle x \rangle)$. Thus we established the relations between higher-order Bernoulli functions and Bernoulli functions. Just as the Fourier series expansion of the Bernoulli functions are useful in computing the special values of Dirichlet *L*-functions, we would like to see some applications to a certain generalization of Dirichlet *L*-functions and higher-order generalized Bernoulli numbers in near future.
